# Prenatal exposure to the Dutch famine is associated with more self-perceived cognitive problems at 72 years of age

**DOI:** 10.1186/s12877-022-02820-2

**Published:** 2022-03-02

**Authors:** Aline Marileen Wiegersma, Amber Boots, Tessa J. Roseboom, Susanne R. de Rooij

**Affiliations:** 1 Department of Epidemiology and Data Science, Amsterdam Public Health research institute, Amsterdam UMC, University of Amsterdam, Meibergdreef 9, 1105 AZ Amsterdam, the Netherlands; 2 Department of Obstetrics and Gynaecology, Amsterdam Public Health research institute, Amsterdam UMC, University of Amsterdam, Meibergdreef 9, Amsterdam, The Netherlands

**Keywords:** Prenatal undernutrition, Cognitive aging, Self-reported

## Abstract

**Background:**

Undernutrition during critical periods of neurodevelopment can hinder the developing brain with lasting negative consequences for brain size, structure and function. In this study, we describe self-perceived cognitive problems of men and women who were born around the time of the Dutch famine of 1944–45.

**Methods:**

We compared self-perceived cognitive problems between men and women who had been exposed to the 1944–45 Dutch famine in late, mid or early gestation and those who were born before or conceived after the famine (and had thus not been exposed prenatally). We included 595 participants aged 71–74 years.

**Results:**

Women who had been exposed to famine in late gestation more often reported cognitive problems compared to those who had not been exposed (OR 2.2 [95% CI 1.1–4.4]), whereas for men, this was the case for those exposed in early gestation (OR 2.3 [0.9–5.5]). Furthermore, men and women exposed in early gestation more often reported consulting a healthcare practitioner for cognitive problems in the past 12 months (OR 3.2 [1.3–8.1]). Especially men exposed in early gestation reported having consulted a healthcare practitioner more often than unexposed men (OR 4.4 [1.2–16.0]).

**Conclusions:**

These findings suggest that prenatal undernutrition does not only have lasting effects on brain size, but also on its function, with more self-perceived cognitive problems at older age, which also require more medical attention. Also, the effects of undernutrition depend on sex and its timing during gestation.

**Supplementary Information:**

The online version contains supplementary material available at 10.1186/s12877-022-02820-2.

## Background

The significance of early-life factors in age-related cognitive decline and risk for dementia is becoming increasingly clear from several studies in humans and animals [1–4]. Adverse events during the prenatal and early postnatal period may directly affect late-life cognitive function and dementia risk by lowering brain and cognitive reserve capacity or by changing the rate at which dementia-related pathologies can accumulate (for instance by limiting the brains’ ability to clear these pathologies) [1, 2]. Indirectly, adverse early-life factors may increase the risk for mediating adverse health outcomes, which in turn have been associated with cognitive decline (e.g. cardiovascular disease) [4–6]. Additionally, adverse early-life factors may accelerate the general aging process and thereby accelerate brain aging [2].

Studies of men and women born around the time of the 1944–45 Dutch famine have provided direct evidence in humans that undernutrition during critical periods of prenatal development increases the risk of age-related diseases such as type 2 diabetes and coronary heart disease, as well as overall mortality [5]. Furthermore, men and women exposed to famine in early gestation performed worse on a selective attention task in their late fifties, which may be an early manifestation of accelerated cognitive aging [6]. Because self-reported cognitive problems may be an indicator of cognitive aging and can be a predictor for future dementia [7], we here describe findings of self-reported cognitive problems at a mean age of 72 years as a further investigation of the effects of prenatal undernutrition on future cognitive decline.

## Methods

### Participants

**Fig. 1:**
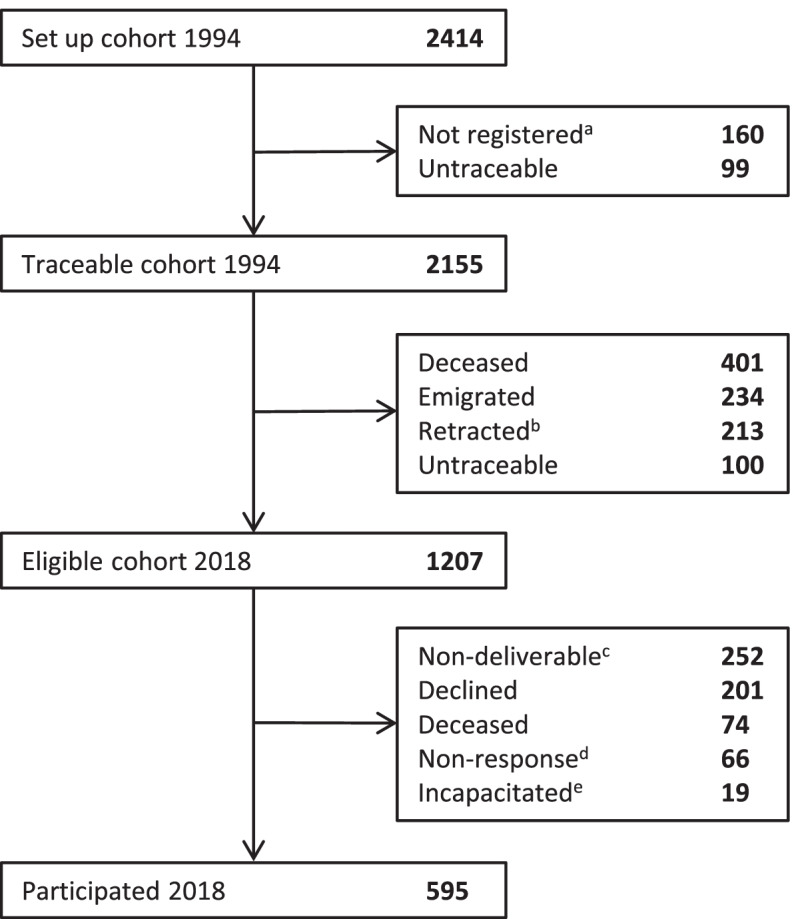
Derivation of study participants. The questionnaire was sent to all 1207 eligible participants. ^a^These babies had not been registered in Amsterdam at birth. ^b^These individuals refused permission to record their address or retracted their permission later. ^c^Invalid home address and could not be reached by phone or no correct phone number and validity of home address unknown. ^d^No telephone contact established (5 attempts) or contact established but no subsequent reaction. ^e^Participants were physically or mentally ill, or cognitively impaired and therefore declined participation

The Dutch famine birth cohort (DFBC) consists of individuals born alive as term singleton babies in the Wilhelmina Gasthuis (a teaching hospital in Amsterdam, the Netherlands) between November 1, 1943 and February 28, 1947. In 1994, 2155 (89.3%) individuals could be included in the DFBC (Fig. 1). In the current study, 1207 individuals were eligible and invited to participate, of which 595 (49.3%) gave written informed consent and completed the questionnaire. The Medical Ethics Review Committee of the Academic Medical Center concluded that a full review and official approval of this study was not required according to Dutch law.

### Exposure

The Dutch famine was a consequence of events occurring at the end of World War II, this cascade of events has been described in detail elsewhere [5, 6]. Daily food rations dropped steeply, reaching a level below 1000 kcal per person on November 26, 1944 and improved relatively quickly after the liberation of the Netherlands in May 1945. There was a more or less proportionate drop of protein, carbohydrate and fat. In correspondence with previous publications on the DFBC, we considered three different 16-week exposure periods: individuals mainly exposed in late gestation (born between January 7 and April 28, 1945), mid-gestation (born between April 29 and August 18, 1945) or early gestation (born between August 19 and December 8, 1945) [6]. Individuals born in these periods were prenatally exposed to at least 13 weeks of famine during which their mother’s daily food-ration contained on average fewer than 1000 ﻿kcal﻿. As children younger than 1 year of age were relatively protected against the famine, both individuals born before (born before January 7, 1945) and conceived after the famine (born after December 8, 1945) were considered unexposed and acted as a control group.

### Self-perceived cognitive problems

Self-perceived cognitive problems were assessed with two questions as part of a larger questionnaire (including mental and physical health, daily life functioning, quality of life and stressful life events). One question was selected from The Older Persons and Informal Caregivers Survey – Minimum DataSet (TOPICS-MDS) validated questionnaire (version before 2017) [8]. Participants had to select the answer that best described their health at that time: “1) I have no problems with my memory, attention and thinking; 2) I have some problems with my memory, attention and thinking; 3) I have severe problems with my memory, attention and thinking.” The second question was adapted from the TOPICS-MDS (version 2017): “Did you consult a doctor or other healthcare practitioner for problems with your memory, attention and thinking in the past 12 months?” [8].

### Study parameters

Maternal**,** pregnancy and birth characteristics were collected from participants’ medical birth records. Hospital anxiety and depression scale (HADS) scores [9], highest achieved education and living situation were derived from the current questionnaire. Socioeconomic status (SES) was derived from individuals’ four-digit current postal code as reported in the questionnaire by matching with Statistics Netherlands (CBS) data. For individuals who had participated in the DFBC studies in 2002 or 2008, we retrieved HADS scores and information about diabetes, stroke and cardiovascular health status at a mean age of 58 and/or 63 years.

### Statistical analyses

We performed logistic regression analyses to investigate associations between famine exposure during late, mid or early gestation and self-perceived cognitive problems, compared to the control groups (Table 2). For each analysis, we ran a crude model and a model adjusted for covariates (sex, birth weight, education and SES). As we have repeatedly observed sex-specific results in previous DFBC-studies, we subsequently performed the analyses separately for men and women. To evaluate potential participation bias we explored differences in birth weight among those not eligible, those eligible participating and those eligible not participating in the current study.

## Results

In total, 134 (22.7%; excluding 4 missing) participants reported to have some problems with their cognition. Only two (0.3%) participants reported severe problems, therefore, we merged some/severe problems in further analyses. Thirty-eight individuals (6.4%; excluding 1 missing) reported to have consulted a healthcare practitioner for cognitive problems in the past 12 months (12 individuals did not report these problems in the first question). Age, sex, birth characteristics, education and SES were not associated with reporting cognitive problems or consulting a healthcare practitioner for these problems (Additional file 1). Individuals who were living alone or scored more than 7 points on either the anxiety or depression scale (either at a mean age of 58, 63 or 72) reported significantly more cognitive problems than individuals who were not living alone or scored 7 or less points on the anxiety or depression scale. The same applied to men and women with hypertension, hypercholesterolemia, vascular problems or diabetes at a mean age of 58 or 63, however, only some of these associations were statistically significant (Additional file 1). Most baseline characteristics were similar, however, some pregnancy, birth and adult characteristics differed among the exposure and control groups (Table 1).

**Table 1:** General, maternal, pregnancy, birth and adult characteristics by timing of prenatal exposure to the Dutch famine

	Exposure to famine						
	N	Born before	In late gestation	In mid gestation	In early gestation	Conceived after	Total
**General characteristics**							
N		183	98	77	50	187	595
N Women (%)	595	96 (52.5)	48 (49.0)	50 (64.9)*	26 (52.0)	89 (47.6)	309 (51.9)
Age (years)	595	73.8 (0.4)***	73.1 (0.1)**	72.8 (0.1)	72.5 (0.1)*	71.9 (0.4)	72.8 (0.8)
**Maternal and pregnancy characteristics**							
Pregnancy duration (days)	520	284 (11)	282 (10)	287 (13)	285 (9.6)	286 (12)	285 (11)
Maternal age at birth (years)	595	28.8 (6.3)	31.1 (6.1)***	29.6 (6.3)	27.0 (5.9)	28.4 (6.2)	29.0 (6.3)
N Primiparous (%)	595	70 (38.3)	19 (19.4)***	23 (29.9)	19 (38.0)	77 (41.2)	208 (35.0)
N Manual occupation head of family (%)	475	108 (79.4)*	52 (59.8)*	49 (74.2)	27 (64.3)	97 (67.4)	333 (70.1)
**Birth characteristics**							
Birth weight (g)	595	3373 (458)	3222 (498)***	3204 (470)***	3503 (377)	3446 (478)	3360 (476)
Head circumference (cm)	588	32.8 (1.4)	32.5 (1.7)**	32.2 (1.4)***	32.9 (1.4)	33.1 (1.6)	32.8 (1.5)
**Adult characteristics**							
2002^a^							
N Hypercholesterolemia (%)	483	46 (30.1)	21 (25.3)	24 (32.9)	11 (29.7)	37 (27.0)	139 (28.8)
N Hypertension (%)	483	49 (32.0)	24 (28.9)	30 (41.1)	10 (27.0)	51 (37.2)	164 (34.0)
N Stroke or TIA (%)	483	0	0	3 (4.1)	0	0	3 (0.6)
N Diabetes type 2 (%)	483	26 (17.0)	12 (14.5)	9 (12.3)	5 (13.5)	14 (10.2)	66 (13.7)
N Vascular problems^c^ (heart infarction or angina pectoris) (%)	483	7 (4.6)	5 (6.0)	4 (5.5)	1 (2.7)	4 (2.9)	21 (4.3)
N HADS anxiety >7^b^ (%)	463	20 (13.4)	10 (13.2)	14 (19.7)	10 (29.4)	29 (21.8)	83 (17.9)
N HADS depression >7^b^ (%)	461	7 (4.7)	5 (6.5)	4 (5.6)	5 (15.2)	12 (9.2)	33 (7.2)
2008^a^							
N Hypercholesterolemia (%)	387	57 (47.1)	16 (24.2)**	27 (47.4)	15 (50.0)	43 (38.1)	158 (40.8)
N Hypertension (%)	388	43 (35.5)	23 (34.3)	27 (47.4)	14 (46.7)	51 (45.1)	158 (40.7)
N Stroke or TIA (%)	388	3 (2.5)	3 (4.5)	3 (5.3)	2 (6.7)	2 (1.8)	13 (3.4)
N Diabetes type 2 (%)	388	16 (13.2)	11 (16.4)	4 (7.0)	6 (20.0)	13 (11.5)	50 (12.9)
N Vascular problems^c^ (heart infarction or angina pectoris) (%)	387	14 (11.6)	5 (7.6)	4 (7.0)	4 (13.3)	11 (9.7)	38 (9.8)
N HADS anxiety >7^b^ (%)	369	17 (15.2)	7 (11.1)	7 (12.3)	8 (30.8)	18 (16.2)	57 (15.4)
N HADS depression >7^b^ (%)	372	7 (6.2)	6 (9.2)	5 (8.9)	7 (25.0)**	9 (8.2)	34 (9.1)
2018							
Level of education^c^	589	4.8 (1.4)	4.9 (1.5)	4.8 (1.3)	4.5 (1.2)	4.9 (1.4)	4.8 (1.4)
Socioeconomic status^d^	590	0.08 (1.02)	0.16 (1.07)	0.13 (0.98)	0.14 (1.05)	0.08 (1.14)	0.10 (1.06)
N Living alone (%)	591	65 (35.5)*	24 (24.7)	23 (29.9)	17 (34.0)	43 (23.4)	172 (29.1)
N HADS anxiety >7^b^ (%)	584	21 (11.8)	11 (11.7)	9 (12.0)	8 (16.0)	32 (17.1)	81 (13.9)
N HADS depression >7^b^ (%)	586	14 (7.8)	6 (6.3)	10 (13.2)	8 (16.0)	17 (9.1)	55 (9.4)

Numbers represent frequencies(%) or means (SD). Asterisks represent *p*-values of linear and logistic regression analyses compared with participants unexposed to famine in gestation, or, for individuals born before the famine, compared to individuals conceived after the famine

*SES* socioeconomic status, *TIA* Transient Ischemic Attack, *HADS* hospital anxiety and depression scale

^a^These data were only available for participants participating at a mean age of 58 (2002) or 63 (2008) in the Dutch Famine Birth Cohort. Hypercholesterolemia, Hypertension and stroke or TIA were derived from questions asking if participants had ever had these health conditions. Diabetes was also based on the questionnaire (2002 and 2008) or having a fasting plasma glucose of ≥7 mmol/l or a 2-h plasma glucose of ≥11.1 mmol/l following a 75 g oral glucose load (2002). Vascular problems were defined as either having a history of heart infarction or probable angina pectoris (based on multiple questions related to chest pain)

^b^Anxiety and depression score > 7 on the hospital anxiety and depression scale (HADS) [9]

^c^Level of education as the highest level of finished schooling measured on a 7-point scale; 1) Less than 6 years of primary school, 2) 6 years of primary school, 3) more than primary school, without an additional diploma, 4) craft school, 5) (pre-)secondary vocational education, 6) pre-university education, 7) higher professional education/university

^d^Based on zip code and data from Statistics Netherlands from 2017 (CBS) (Range: −4.76-2.78, mean: 0.10 in our dataset)

* < 0.05, ** < 0.01, *** < 0.001

Overall, the prevalence of reporting cognitive problems did not differ between those unexposed and those prenatally exposed to famine (Table 2). However, those exposed in early gestation more often reported having consulted a healthcare practitioner for cognitive problems in the past 12 months than controls (OR: 3.2 [95% CI 1.3–8.1]). Looking at men and women separately showed that women exposed in late gestation more often reported cognitive problems (OR 2.2 [1.1–4.4]), whereas men exposed in early gestation more often reported cognitive problems (OR 2.3 [0.9–5.5]) than those who had not been exposed to famine prenatally. Men exposed in early gestation four times more often reported having consulted a healthcare practitioner for cognitive problems than men who had not been exposed prenatally (OR 4.4 [1.2–16.0]). Adjusting for covariates did not attenuate the estimates.

**Table 2:** Association of prenatal exposure to famine with self-reported cognitive problems compared to individuals born before or conceived after the famine

		Exposure to famine			
	Born Before	In late gestation	In mid gestation	In early gestation	Conceived after
**Question 1:** I have some/severe problems with my memory, attention and thinking					
**Full Cohort:** N (%)	41 (22.5)	21 (21.4)	22 (28.6)	15 (30.0)	37 (20.1)
OR (95% CI) Model 1^a^	Reference	1.0 (0.6–1.7)	1.5 (0.8–2.6)	1.6 (0.8–3.1)	Reference
OR (95% CI) Model 2^b^	Reference	1.1 (0.6–1.9)	1.7 (0.9–2.9)	1.7 (0.9–3.3)	Reference
**Women:** N (%)	18 (18.8)	16 (33.3)	12 (24.0)	5 (19.2)	16 (18.6)
OR (95% CI) Model 3^c^	Reference	**2.2 (1.1–4.4)**	1.4 (0.7–2.9)	1.0 (0.4–2.9)	Reference
OR (95% CI) Model 4^d^	Reference	**2.5 (1.2–5.4)**	1.6 (0.7–3.4)	1.1 (0.4–3.4)	Reference
**Men:** N(%)	23 (26.7)	5 (10.0)	10 (37.0)	10 (41.7)	21 (21.4)
OR (95% CI) Model 3^c^	Reference	0.4 (0.1–0.9)	1.9 (0.8–4.4)	2.3 (0.9–5.5)	Reference
OR (95% CI) Model 4^d^	Reference	0.4 (0.1–1.0)	2.0 (0.8–4.8)	2.2 (0.9–5.7)	Reference
**Question 2:** I consulted a doctor or other healthcare practitioner for problems with your memory, attention and thinking in the past 12 months					
**Full Cohort:** N (%)	9 (4.9)	8 (8.2)	5 (6.6)	7 (14.0)	9 (4.8)
OR (95% CI) Model 1^a^	Reference	1.7 (0.7–4.1)	1.4 (0.5–3.8)	**3.2 (1.3–8.1)**	Reference
OR (95% CI) Model 2^b^	Reference	1.9 (0.8–4.6)	1.3 (0.5–3.8)	**2.9 (1.1–7.5)**	Reference
**Women:** N (%)	5 (5.2)	4 (8.3)	4 (8.0)	3 (11.5)	21 (6.8)
OR (95% CI) Model 3^c^	Reference	1.6 (0.5–5.3)	1.5 (0.5–5.1)	2.3 (0.6–8.9)	Reference
OR (95% CI) Model 4^d^	Reference	1.8 (0.5–6.2)	1.4 (0.4–4.7)	2.4 (0.6–9.7)	Reference
**Men:** N (%)	4 (4.7)	4 (8.0)	1 (3.8)	4 (16.7)	4 (4.1)
OR (95% CI) Model 3^c^	Reference	1.9 (0.6–6.7)	0.9 (0.1–7.4)	**4.4 (1.2–16.0)**	Reference
OR (95% CI) Model 4^d^	Reference	2.6 (0.7–9.5)	1.0 (0.1–8.4)	**4.0 (1.0–15.5)**	Reference

Significant (*p* < 0.05) findings are depicted in bold

*SES* socioeconomic status

^a^Model 1: Logistic regression model, unadjusted

^b^Model 2: Logistic regression model, adjusted for sex, birth weight, education and SES

^c^Model 3: Sex-specific logistic regression model, unadjusted

## Discussion

Our findings suggest that prenatal undernutrition increases the risk of cognitive problems in later life, which also leads to more often consulting a healthcare practitioner for these problems. This increased risk appears to depend on sex and timing during gestation. Particularly men who had been exposed to famine in early gestation more often reported cognitive problems and consulting a healthcare practitioner for these problems. This is in line with the poorer cognitive function in their late fifties [5]. Interestingly, women exposed to famine in late gestation more often reported cognitive problems.

### Agreement with other studies

To our knowledge, no other studies have investigated associations between prenatal undernutrition and self-perceived cognitive problems later in life. Studies regarding the Chinese Great Leap Forward famine of 1959–61 did show that men and women prenatally exposed to undernutrition had more cognitive impairments (measured with cognitive tests) and dementia than controls [10, 11]. However, most Chinese famine studies are hampered by only selecting controls born after the famine, thereby not accounting for age differences (a major risk factor) and potentially overestimating their results. In the DFBC, we account for this bias by also including controls born before the famine. Furthermore, studies have linked other adverse prenatal and early-life circumstances to late-life cognitive problems and dementia [1–4, 6]. Good economic conditions at the time of birth have for instance been associated with a higher chance of good cognitive functioning at age 60 and older, while a recession was associated with lower cognitive functioning at those ages [12]. Only one study investigated, and found, an association between a prenatal exposure (maternal hypertensive disorder) and subjective cognitive complaints [13].

### Interpretation and potential mechanisms

In previous DFBC-studies, men exposed in early gestation were shown to have smaller total brain volume and increased BrainAGE at age 68 [14, 15]. These findings may point at a compromised brain reserve, which may result in an earlier crossing of the threshold below which normal cognitive functioning cannot be sustained [4]. Furthermore, animal studies have indicated that early-life circumstances can modify the rate of accumulation of dementia-related pathologies [2]. Additionally, as hypothesized previously [6], our current observations could be due to vascular effects. Prior DFBC-studies showed increased risks for cardiovascular and metabolic diseases in exposed individuals, as well as overall worse brain perfusion [5, 16]. Building on this, the association we observed is likely not a direct effect alone. We lacked statistical power to formally explore mediators, however, we observed more reporting of cognitive problems in individuals with hypercholesterolemia or hypertension around age 58 and 63 in our cohort. Furthermore, high anxiety and depression scores measured at a mean age of 58, 63 and 72 were strongly associated with self-perceived cognitive problems. Anxiety and depression symptoms seem more prevalent among men and women who had been exposed in early gestation compared to controls, similar to what we have observed before in men exposed in early gestation [17]. Associations with anxiety and depression symptoms reported 10 years earlier would suggest a possible role for these symptoms as mediating factors. Some studies have indicated that earlier depressive episodes may confer increased risk for later pathologic cognitive decline [18]. However, the relationship between depression and anxiety - especially in older adults - and self-perceived cognitive problems is complex [7]. Anxiety and depression may influence the awareness and occurrence of cognitive problems, yet, anxiety and depression in older adults have been associated with objective measures of brain aging and increased risk for dementia [7].

There are differences in the prenatal development of men and women, men are, for instance, more vulnerable to certain adverse prenatal circumstances [19]. In earlier studies in the same cohort, we have observed sex-specific effects similar to the ones described in the current study, the effects of exposure to famine in early gestation were more pronounced in men than in women. These results could be due to the increased vulnerability of men due to their early life growth strategy [20]). Alternatively, excess mortality in women exposed early in gestation, may have led to an underestimation of the effect in women as relatively healthier women remain [21].

### Strengths and limitations

Strengths of the current study include that the results are in line with previous findings and seem relatively robust. We made use of a unique cohort with a quasi-experimental set up, which limits the risks of bias and confounding [5, 6].

This study has limitations. The number of participants was small, thus we had limited power in our analyses, especially regarding consulting a healthcare practitioner for cognitive problems and the sex-stratified analyses. A limitation of the current study is the lack of objective information about cognitive status and/or dementia diagnosis in the cohort. Regardless, we do observe significant associations. Furthermore, only 50.0% of the initial cohort was eligible to participate in 2018, and of this group, 49.3% participated in the current study. Birth weights were similar among these groups, however, adult characteristics may differ and selective participation may have caused bias. Individuals with (early stage) dementia may, for instance, be less likely to participate in questionnaires, and when they do, may fail to recognize their cognitive problems [7]. However, as our study sample is relatively healthy and relatively young we expect to mainly see early and less severe symptoms of cognitive decline. As prenatal famine exposure has previously been associated with poorer health outcomes, we expect a relatively greater loss of participants with poorer health in the exposed groups [22]. Both participation bias and possible misrepresentation of dementia diagnoses would most likely lead to an underestimation of the associations, this may especially be true for people who had been exposed in early gestation who seemed most affected by brain aging and health problems [5, 6, 14–16]. Furthermore, adding covariates to the models did not substantially attenuate our estimates, which adds to the robustness of our findings, however, the possibility of residual confounding remains.

Regarding the exposure, we cannot be absolutely sure that our associations are solely due to prenatal undernutrition. Maternal stress due to the food scarcity and war conditions may have also played a role [23]. The stress experienced by pregnant women during the famine was likely more extreme than the stress experienced by women before or after the famine [23]. Regarding the outcome, the current study focused on a subjective measure of cognitive aging. Although objective measures of cognitive and brain aging remain the gold standard, self-perceived cognitive problems are of importance. Self-perceived cognitive problems usually occur before any other objective measures of cognitive problems and can be predictive for future objective cognitive decline and the development of mild cognitive impairment (MCI) and dementia [7, 24]. Studies have suggested that self-perceived cognitive problems represent one of the earliest symptoms of Alzheimer’s disease [7, 24]. Furthermore, subjective cognitive problems have been associated with measures of brain aging, like hippocampal atrophy and greater white matter hyperintensity volume [25]. Self-perceived cognitive problems, in our sample, show - to be expected - associations with measures of depression and anxiety, strengthening our confidence in their validity [7]. As described earlier the relationship between self-perceived cognitive problems and anxiety and depression is complex. One study showed associations between self-perceived cognitive problems and more objective cognitive decline and higher risk of MCI and dementia at the end of follow-up, while excluding participants with clinical depression [24]. These findings would indicate that the predictive value of self-perceived cognitive problems is not solely dependent on the presence of depressive symptoms.

Interestingly, 12 individuals reported to have consulted a healthcare practitioner for cognitive problems while they did not report having cognitive problems. Possibly, this discrepancy is due to others worrying about the participants’ cognitive health. Excluding these individuals from the analyses regarding consulting a healthcare practitioner led to slightly higher effect estimates regarding the association between prenatal undernutrition and self-perceived cognitive problems, suggesting we might underestimate our findings by including these individuals.

## Conclusion

Our results suggest that prenatal undernutrition increases the risk of self-perceived cognitive problems in men and women, and that this effect depends on sex and the timing during gestation. Possibly, these self-perceived cognitive problems are predictive of further cognitive decline and dementia [7]. Future studies will investigate dementia prevalence in these men and women as they age.

## Supplementary Information

**Additional file 1.** Association of covariates with self-perceived cognitive problems. Description: We explored associations between covariates and self-perceived cognitive problems with logistic regression analyses.

## Data Availability

The datasets supporting the conclusion of this article are not available in a public repository. Data can be made available by the corresponding author upon reasonable request.

## Abbreviations

DFBC: Dutch Famine Birth Cohort
CI: Confidence Interval
HADS: Hospital Anxiety and Depression Scale
OR: Odds Ratio
SES: Socioeconomic Status
TIA: Transient Ischemic Attack
TOPICS-MDS: The Older Persons and Informal Caregivers Survey – Minimum DataSet
