# Correction: Prenatal exposure to the Dutch famine is associated with more self-perceived cognitive problems at 72 years of age

**DOI:** 10.1186/s12877-022-03023-5

**Published:** 2022-05-10

**Authors:** Aline Marileen Wiegersma, Amber Boots, Tessa J. Roseboom, Susanne R. de Rooij

**Affiliations:** 1 Department of Epidemiology and Data Science, Amsterdam Public Health Research Institute, Amsterdam UMC, University of Amsterdam, Meibergdreef 9, 1105 AZ Amsterdam, the Netherlands; 2 Department of Obstetrics and Gynaecology, Amsterdam Public Health Research Institute, Amsterdam UMC, University of Amsterdam, Meibergdreef 9, Amsterdam, The Netherlands

**Correction to: BMC Geriatrics 22, 176 (2022)**

**https://doi.org/10.1186/s12877-022-02820-2**

After publication of this article [1], the authors reported some minor errors in Tables 1 and 2.

- In Table 1, there is a superfluous c behind “N Vascular problems”; this can be removed twice.
- In Table 1, “Exposure to famine” is aligned incorrectly; should be moved to the right, above “In late gestation; In mid gestation; In early gestation” (as in Table 2).
- In Table 2, a note is missing: “d Model 4: Sex-specific logistic regression model, adjusted for birth weight, education and SES”

The original article [1] has been updated.
