# Supplementary material for: Prenatal exposure to the Dutch famine is associated with more self-perceived cognitive problems at 72 years of age
Source: BMC Geriatr. 2022 Mar 2;22:176. doi: 10.1186/s12877-022-02820-2 (PMC8892724; doi:10.1186/s12877-022-02820-2)
Supplement: Supplementary file 1 — Additional file 1. Association of covariates with self-perceived cognitive problems. Description: We explored associations between covariates and self-perceived cognitive problems with logistic regression analyses. [file 12877_2022_2820_MOESM1_ESM.docx]

**Additional file 1.** Association of covariates with self-perceived cognitive problems

|  | | | | | | | | | | | | | |
| --- | --- | --- | --- | --- | --- | --- | --- | --- | --- | --- | --- | --- | --- |
|  | | | I have some/severe problems with my memory, attention and thinking | | | | | | I consulted a doctor or other healthcare practitioner for problems with your memory, attention and thinking in the past 12 months | | | | |
|  | | | Yes | | No | | OR (95% CI)^a^ | | Yes | | No | | OR (95% CI)^a^ |
| **General characteristics** | | | | | | | | | | | | | |
|  | Sex | | | | | | | | | | | | |
|  | | Women | 67 (21.9) | | 239 (78.1) | | 0.9 (0.6-1.3) | | 21 (6.8) | | 288 (93.2) | | 1.2 (0.6-2.2) |
|  | | Men | 69 (24.2) | | 216 (75.8) | | Reference | | 17 (6.0) | | 268 (94.0) | | Reference |
|  | Age (years) | | | | | | | | | | | | |
|  | | | 72.9 (0.8) | | 72.8 (0.8) | | 1.0 (0.8-1.3) | | 72.81 (0.71) | | 72.8 (0.8) | | 1.0 (0.6-1.4) |
| **Birth characteristics** | | | | | | | | | | | | | |
|  | Birth weight (g) | | | | | | | | | | | | |
|  | | | 3348 (493) | | 3365 (466) | | 1.0 (1.0-1.0) | | 3441 (476) | | 3355 (476) | | 1.00 (1.00-1.00) |
|  | Gestational age (days) | | | | | | | | | | | | |
|  | | | 286 (12) | | 285 (11) | | 1.0 (1.0-1.0) | | 286 (12) | | 285 (11) | | 1.0 (1.0-1.0) |
|  | Head circumference (cm) | | | | | | | | | | | | |
|  | | | 32.7 (1.5) | | 32.8 (1.5) | | 1.0 (0.9-1.1) | | 32.8 (1.6) | | 32.8 (1.5) | | 1.0 (0.8-1.2) |
| **Adult characteristics** | | | | | | | | | | | | | |
| **2002^e^** | | | | | | | | | | | | | |
|  | Hypercholesterolemia | | | | | | | | | | | | |
|  | | Yes | 46 (33.1) | | 93 (66.9) | | 1.9 (1.2-2.9) | | 11 (8.0) | | 127 (92.0) | | 1.2 (0.6-2.6) |
|  | | No | 72 (21.1) | | 270 (78.9) | | Reference | | 23 (6.7) | | 321 (93.3) | | Reference |
|  | Hypertension | | | | | | | | | | | | |
|  | | Yes | 40 (24.4) | | 124 (75.6) | | 1.0 (0.6-1.5) | | 17 (10.4) | | 147 (89.6) | | 2.0 (1.0-4.1) |
|  | | No | 78 (24.6) | | 239 (75.4) | | Reference | | 17 (5.3) | | 301 (94.7) | | Reference |
|  | Stroke or TIA | | | | | | | | | | | | |
|  | | Yes | 1 (33.3) | | 2 (66.7) | | 1.5 (0.1-17.2) | | 3 (100.0) | | 0 (0.0) | | - |
|  | | No | 117 (24.5) | | 361 (75.5) | | Reference | | 34 (7.1) | | 445 (92.9) | | Reference |
|  | Diabetes type 2 | | | | | | | | | | | | |
|  | | Yes | 15 (23.1) | | 50 (76.9) | | 0.9 (0.5-1.7) | | 7 (10.6) | | 59 (89.4) | | 1.7 (0.7-4.1) |
|  | | No | 103 (24.8) | | 313 (75.2) | | Reference | | 27 (6.5) | | 389 (93.5) | | Reference |
|  | Vascular problems (heart infarction or angina pectoris) | | | | | | | | | | | | |
|  | | Yes | 9 (42.9) | | 12 (57.1) | | 2.4 (1.0-5.9) | | 1 (4.8) | | 20 (95.2) | | 0.6 (0.1-5.0) |
|  | | No | 109 (23.7) | | 351 (76.3) | | Reference | | 33 (7.2) | | 428 (92.8) | | Reference |
|  | HADS anxiety >7^d^ | | | | | | | | | | | | |
|  | | Yes | 34 (41.5) | | 48 (58.5) | | 2.7 (1.7-4.5) | | 15 (18.1) | | 68 (81.9) | | 4.7 (2.2-9.9) |
|  | | No | 78 (20.6) | | 301 (79.4) | | Reference | | 17 (4.5) | | 362 (95.5) | | Reference |
|  | HADS depression >7^d^ | | | | | | | | | | | | |
|  | | Yes | 14 (43.8) | | 18 (56.3) | | 2.6 (1.3-5.4) | | 5 (15.2) | | 28 (84.8) | | 2.5 (0.9-7.1) |
|  | | No | 98 (23.0) | | 329 (77.0) | | Reference | | 28 (6.6) | | 399 (93.4) | | Reference |
| **2008^e^** | | | | | | | | | | | | | |
|  | Hypercholesterolemia | | | | | | | | | | | | |
|  | | Yes | 48 (30.6) | | 109 (69.4) | | 1.7 (1.1-2.8) | | 16 (10.2) | | 141 (89.8) | | 2.8 (1.2-6.5) |
|  | | No | 46 (20.2) | | 182 (79.8) | | Reference | | 9 (3.9) | | 220 (96.1) | | Reference |
|  | Hypertension | | | | | | | | | | | | |
|  | | Yes | 41 (25.9) | | 117 (74.1) | | 1.1 (0.7-1.8) | | 17 (10.8) | | 141 (89.2) | | 3.3 (1.4-7.9) |
|  | | No | 54 (23.7) | | 174 (76.3) | | Reference | | 8 (3.5) | | 221 (96.5) | | Reference |
|  | |  | I have some/severe problems with my memory, attention and thinking | | | | | | I consulted a doctor or other healthcare practitioner for problems with your memory, attention and thinking in the past 12 months | | | | |
|  | |  | Yes | | No | | OR (95% CI)^a^ | | Yes | | No | | OR (95% CI)^a^ |
|  | Stroke or TIA | | | | | | | | | | | | |
|  | | Yes | 6 (46.2) | | 7 (53.8) | | 2.7 (0.9-8.4) | | 3 (23.1) | | 10 (76.9) | | 4.8 (1.2-18.7) |
|  | | No | 89 (23.9) | | 284 (76.1) | | Reference | | 22 (5.9) | | 352 (94.1) | | Reference |
|  | Diabetes type 2 | | | | | | | | | | | | |
|  | | Yes | 14 (28.6) | | 35 (71.4) | | 1.3 (0.6-2.5) | | 5 (10.0) | | 45 (90.0) | | 1.8 (0.6-4.9) |
|  | | No | 81 (24.0) | | 256 (76.0) | | Reference | | 20 (5.9) | | 317 (94.1) | | Reference |
|  | Vascular problems (heart infarction or angina pectoris) | | | | | | | | | | | | |
|  | | Yes | 13 (34.2) | | 25 (65.8) | | 1.7 (0.8-3.5) | | 5 (13.2) | | 33 (86.8) | | 2.5 (0.9-7.1) |
|  | | No | 81 (23.3) | | 266 (76.7) | | Reference | | 20 (5.7) | | 328 (94.3) | | Reference |
|  | HADS anxiety >7^d^ | | | | | | | | | | | | |
|  | | Yes | 23 (40.4) | | 34 (59.6) | | 2.4 (1.3-4.4) | | 9 (15.8) | | 48 (84.2) | | 3.5 (1.4-8.3) |
|  | | No | 68 (21.9) | | 242 (78.1) | | Reference | | 16 (5.1) | | 295 (94.9) | | Reference |
|  | HADS depression >7^d^ | | | | | | | | | | | | |
|  | | Yes | 16 (47.1) | | 18 (52.9) | | 3.0 (1.5-6.3) | | 8 (23.5) | | 26 (76.5) | | 5.8 (2.3-14.7) |
|  | | No | 76 (22.6) | | 260 (77.4) | | Reference | | 17 (5.0) | | 320 (95.0) | | Reference |
| **2018** | |  |  |  | |  | |  | |  | |  | |
|  | Level of education^b^ | | | | | | | | | | | | |
|  | |  | 4.7 (1.4) | | 4.9 (1.4) | | 0.9 (0.8-1.1) | | 4.6 (1.3) | | 4.8 (1.4) | | 0.9 (0.7-1.1) |
|  | SES^c^ | | | | | | | | | | | | |
|  | | | 0.04 (1.11) | | 0.12 (1.05) | | 0.9 (0.8-1.1) | | 0.2 (1.1) | | 0.10 (1.06) | | 1.1 (0.8-1.5) |
|  | Living alone | | | | | | | | | | | | |
|  | | Yes | 50 (29.2) | | 121 (70.8) | | 1.6 (1.1-2.4) | | 16 (9.3) | | 156 (90.7) | | 1.8 (0.9-3.6) |
|  | | No | 86 (20.5) | | 333 (79.5) | | Reference | | 22 (5.3) | | 396 (94.7) | | Reference |
|  | HADS anxiety >7^d^ | | | | | | | | | | | | |
|  | | Yes | 39 (48.8) | | 41 (51.2) | | 4.1 (2.5-6.7) | | 18 (22.2) | | 63 (77.6) | | 7.3 (3.6-14.6) |
|  | | No | 94 (18.8) | | 406 (81.2) | | Reference | | 19 (3.8) | | 483 (96.2) | | Reference |
|  | HADS depression >7^d^ | | | | | | | | | | | | |
|  | | Yes | 30 (55.6) | | 24 (44.4) | | 5.1 (2.9-9.1) | | 11 (20.0) | | 44 (80.0) | | 4.8 (2.2-10.5) |
|  | | No | 104 (19.7) | | 424 (80.3) | | Reference | | 26 (4.9) | | 504 (95.1) | | Reference |

We explored associations between covariates and self-perceived cognitive problems with logistic regression analyses. Numbers are mean (SD) for continuous variables and N (%) for dichotomous variables.

SES = socioeconomic status; TIA = Transient Ischemic Attack; HADS = hospital anxiety and depression scale.

^a^Logistic regression model, unadjusted.

^b^Level of education as the highest level of finished schooling measured on a 7-point scale; 1) Less than six years of primary school, 2) six years of primary school, 3) more than primary school, without an additional diploma, 4) craft school, 5) (pre-)secondary vocational education, 6) pre-university education, 7) higher professional education/university.

^c^Based on zip code and data from Statistics Netherlands from 2017 (CBS) (Range: -4.76-2.78, mean: 0.10 in our dataset).

^d^Anxiety and depression score >7 of the hospital anxiety and depression scale (HADS) (9).

^e^These data were only available for participants participating at a mean age of 58 (2002) or 63 (2008) in the Dutch Famine Birth Cohort. Hypercholesterolemia, Hypertension and stroke or TIA were derived from questions asking if participants had ever had these health conditions. Diabetes was also based on the questionnaire (2002 and 2008) or having a fasting plasma glucose of ≥7 mmol/l or a 2-hour plasma glucose of ≥11.1 mmol/l following a 75g oral glucose load (2002). Vascular problems were defined as either having a history of heart infarction or probable angina pectoris (based on multiple questions related to chest pain).
